# Genome wide linkage disequilibrium and genetic structure in Sicilian dairy sheep breeds

**DOI:** 10.1186/s12863-014-0108-5

**Published:** 2014-10-10

**Authors:** Salvatore Mastrangelo, Rosalia Di Gerlando, Marco Tolone, Lina Tortorici, Maria Teresa Sardina, Baldassare Portolano, International Sheep Genomics Consortium

**Affiliations:** Dipartimento Scienze Agrarie e Forestali, Università degli Studi di Palermo, Viale delle Scienze, 90128 Palermo, Italy

**Keywords:** OvineSNP50K BeadChip, Sicilian sheep breeds, Linkage disequilibrium, Genome structure

## Abstract

**Background:**

The recent availability of sheep genome-wide SNP panels allows providing background information concerning genome structure in domestic animals. The aim of this work was to investigate the patterns of linkage disequilibrium (LD), the genetic diversity and population structure in Valle del Belice, Comisana, and Pinzirita dairy sheep breeds using the Illumina Ovine SNP50K Genotyping array.

**Results:**

Average *r*^*2*^ between adjacent SNPs across all chromosomes was 0.155 ± 0.204 for Valle del Belice, 0.156 ± 0.208 for Comisana, and 0.128 ± 0.188 for Pinzirita breeds, and some variations in LD value across chromosomes were observed, in particular for Valle del Belice and Comisana breeds. Average values of *r*^*2*^ estimated for all pairwise combinations of SNPs pooled over all autosomes were 0.058 ± 0.023 for Valle del Belice, 0.056 ± 0.021 for Comisana, and 0.037 ± 0.017 for Pinzirita breeds. The LD declined as a function of distance and average *r*^*2*^ was lower than the values observed in other sheep breeds. Consistency of results among the several used approaches (Principal component analysis, Bayesian clustering, *F*_ST,_ Neighbor networks) showed that while Valle del Belice and Pinzirita breeds formed a unique cluster, Comisana breed showed the presence of substructure. In Valle del Belice breed, the high level of genetic differentiation within breed, the heterogeneous cluster in Admixture analysis, but at the same time the highest inbreeding coefficient, suggested that the breed had a wide genetic base with inbred individuals belonging to the same flock. The Sicilian breeds were characterized by low genetic differentiation and high level of admixture. Pinzirita breed displayed the highest genetic diversity (He, N_e_) whereas the lowest value was found in Valle del Belice breed.

**Conclusions:**

This study has reported for the first time estimates of LD and genetic diversity from a genome-wide perspective in Sicilian dairy sheep breeds. Our results indicate that breeds formed non-overlapping clusters and are clearly separated populations and that Comisana sheep breed does not constitute a homogenous population. The information generated from this study has important implications for the design and applications of association studies as well as for development of conservation and/or selection breeding programs.

**Electronic supplementary material:**

The online version of this article (doi:10.1186/s12863-014-0108-5) contains supplementary material, which is available to authorized users.

## Background

The application of recently developed genomic technology, such as high-density single nucleotide polymorphism (SNP) arrays, has great potential to increase our understanding on the genetic architecture of complex traits, to improve selection efficiency in domestic animals through genomic selection [1], and to conduct association studies [2]. However, to optimally plan whole-genome association studies, it is crucial to know the extent of linkage disequilibrium (LD), the non-random association of alleles at different loci in the genome. In fact, the extent of LD is often used to determine the optimal number of markers required for fine mapping of quantitative trait loci (QTL) [3], for genomic selection [4], and to understand the evolutionary history of the populations [5]. With this in mind, it is important to quantify the extent of LD within different breeds as this is likely to have an impact on the success of gene mapping experiments [6]. Knowledge concerning the extent of genetic diversity, as the level of inbreeding and population structure is critical for each of these applications [7]. Moreover, effective population size (N_e_) is an important parameter for the assessment of genetic diversity within a livestock population and its development over time. If pedigree information is not available, LD analysis might offer an alternative perspective for the estimation of N_e_ [5]. In local breeds, maintaining genetic variability is an important requirement for animal breeding strategies; this guarantees selection response to productive and adaptive traits improvement, to cope with new environmental conditions, changes in market demands, husbandry practices and disease challenges [8]. Currently, with availability of high-density SNP arrays, genetic diversity can be estimated accurately in the absence of pedigree information [9].

In Sicily, dairy sheep production represents an important resource for the economy of hilly and mountain areas, in which other economic activities are limited [10]. Nowadays, three native dairy sheep breeds are reared in Sicily: Valle del Belice, Comisana and Pinzirita. These breeds present differences both in morphology and production traits, showing excellent adaptability to local environments. The aim of this work was to investigate these breeds from a genetic perspective, including the analysis of: i) genome-wide levels of LD, ii) genetic diversity and population structure using high-density genotyping arrays.

## Results and discussion

In the present study, the Illumina OvineSNP50K Genotyping BeadChip was used to characterize LD and to analyze genetic diversity and population structure in Sicilian dairy sheep breeds.

Out of a total of 54,241 SNPs genotyped, 378 were unmapped and 1,450 were located on sex chromosomes. Thus, 52,413 SNPs mapped onto 26 sheep autosomes were used, and after filtering (see Methods), the final number of samples and SNPs were 71 and 44,365 for Valle del Belice, 71 and 44,540 for Comisana, and 77 and 45,451 for Pinzirita sheep breeds. The distribution of SNPs per chromosome and breed was reported in Additional file 1: Table S1.

### Linkage Disequilibrium (LD)

**Figure 1:**
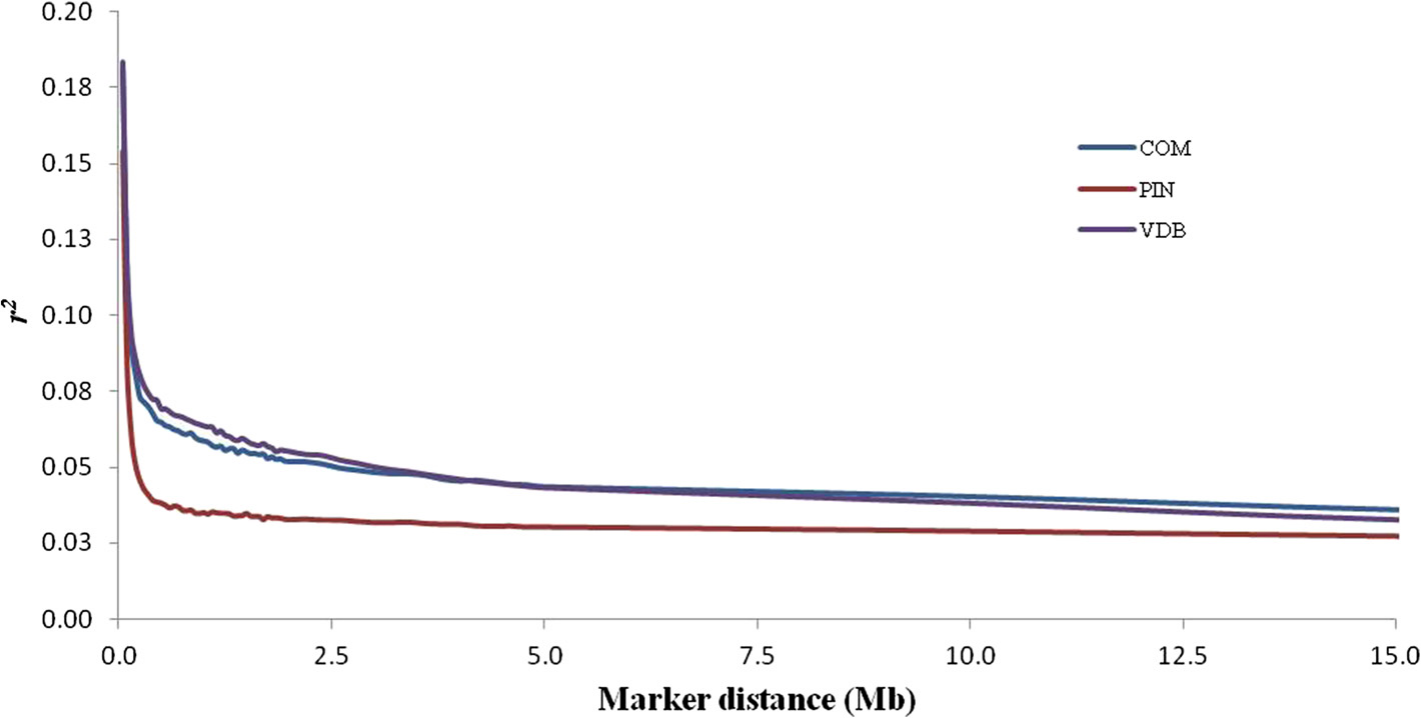
**Linkage disequilibrium across the genome as a function of genomic distance.** Valle del Belice (VDB), Comisana (COM) and Pinzirita (PIN) breeds.

The extent of LD was first evaluated for each adjacent SNP pairs. The *r*^*2*^ was used as measure of LD, because is the most suitable measure of LD for biallelic markers [11] and to avoid the influence of small sample size [12]. The average distances between adjacent SNP pairs for the whole autosomal genome were about 60 kb for Valle del Belice and Comisana, and 59 kb for Pinzirita sheep breeds (Table 1). The *r*^*2*^ ranged from 0.133 ± 0.187 for OAR14 to 0.182 ± 0.229 for OAR2 in Valle del Belice, from 0.134 ± 0.188 for OAR14 to 0.189 ± 0.237 for OAR2 in Comisana, whereas the lowest values among chromosomes were observed in Pinzirita, where *r*^*2*^ ranged from 0.107 ± 0.164 for OAR23 to 0.148 ± 0.208 for OAR2 (Table 1). Mean values of *r*^*2*^ estimated for all pairwise SNPs combinations pooled over all autosomes were 0.058 ± 0.023 for Valle del Belice, 0.056 ± 0.021 for Comisana, and 0.037 ± 0.017 for Pinzirita sheep breeds. In order to examine the decay of LD with physical distance, SNP pairs on autosomes were sorted into bins based on their inter-marker distance and average values of *r*^*2*^ were calculated for each bin. Pairwise *r*^*2*^ values were also averaged over all autosomes and plotted as a function of genomic distance between markers. Levels of pairwise LD decreased with increasing distance between SNPs, as reported in Figure 1 and Additional file 2: Table S2. For SNPs up to 50 kb apart the average *r*^*2*^ was 0.183, 0.181, and 0.154, for SNPs separated by 200–500 kb the average *r*^*2*^ was 0.074, 0.067, and 0.041, and when SNPs were separated by more than 2,000 kb the average *r*^*2*^ was 0.041, 0.042 and 0.029 in Valle del Belice, Comisana, and Pinzirita sheep breeds, respectively. These results could be attributed to recombination rate varying between and within chromosomes, differences in chromosome length, heterozygosity, genetic drift, and effect of selection [13]. Effect of selection on LD is dependent upon direction, intensity, duration, and consistency of selection over time. In fact, Pinzirita breed is not subject to breeding programs, while Comisana and Valle del Belice breeds are characterized by low selection pressure. The comparison of LD levels obtained in different studies is not straightforward, because of differences in several factors such as sample size, type of LD measures (*D’* or *r*^*2*^), marker types (microsatellite or SNP), marker density and distribution, and population demography [13]. Moreover, so far, results of the extent of LD have been reported for wide-spread and important sheep breeds, and there is little knowledge about the degree of genome-wide LD in local sheep breeds. García-Gámez et al. [1] in a study on LD in Spanish Churra sheep breed reported an average *r*^*2*^ estimated for all pairwise combinations per chromosomes that ranged from 0.006 in OAR1 to 0.015 in OAR20, and an average *r*^*2*^ of 0.061 for SNPs separated by 200–500 kb, in agreement with our results. Usai et al. [14] in a study of LD in a sample of Sarda rams showed higher value with an average *r*^*2*^ over 1,000 kb of 0.072. Previous studies in five populations of domestic sheep based on microsatellite markers [6] and in wild sheep based on dense panel of SNPs [15] showed LD extends over long distance (*r*^*2*^ = 0.192 ± 0.131 for markers separated by 5–10 cM, and high levels of LD observed over 4 Mb, respectively). Levels of *r*^*2*^ for adjacent SNP pairs and all pairwise combinations of SNPs in Sicilian sheep breeds were lower than values observed in other livestock species such as pig [16,17], cattle [13,18], and horse [19,20]. These differences can be explained considering the intensive artificial selection to which commercial animal breeding populations (pig, cattle and horse) have been subjected for many generations and the ensuing reduction in effective population size. In fact, these species share a similar mating system using popular sires, whereas in ovine species, and in particular in Sicilian farming system, natural mating is the common practice. The decay of LD in a genome determines the power of QTL detection in association mapping studies and helps to determine the number of markers required for successful association mapping and genomic selection. Meuwissen et al. [4], in a simulation to predict genomic breeding values from dense markers across the whole genome with accuracies up to 0.85, found a required *r*^*2*^ level of 0.2. Qanbari et al. [13] considered *r*^*2*^ threshold of 0.25 as a useful LD value for association studies. In fact, species with extensive LD will require fewer markers than those with low levels of LD. Therefore, these results support the need to use more dense SNP panels for high power association mapping and genomic selection efficiency in future breeding programs for Sicilian dairy sheep breeds, and in particular for Pinzirita breed.

**Table 1:** **Average space (bp), Linkage Disequilibrium (** ***r*** ^***2***^ **) and standard deviation (s.d.) between adjacent single nucleotide polymorphisms (SNPs) on each chromosome (OAR) in the Sicilian sheep breeds**

	**VDB**		**COM**		**PIN**	
**OAR**	**Average space**	**r** ^**2**^ **± s.d.**	**Average space**	**r** ^**2**^ **± s.d.**	**Average space**	**r** ^**2**^ **± s.d.**
1	60,381	0.162 ± 0.205	60,211	0.161 ± 0.213	58,874	0.132 ± 0.190
2	56,594	0.182 ± 0.229	57,023	0.189 ± 0.237	55,298	0.148 ± 0.208
3	57,120	0.170 ± 0.217	56,744	0.172 ± 0.224	55,389	0.140 ± 0.198
4	55,435	0.171 ± 0.219	54,484	0.164 ± 0.216	53,770	0.139 ± 0.200
5	57,306	0.150 ± 0.199	57,292	0.154 ± 0.205	55,749	0.127 ± 0.188
6	57,242	0.158 ± 0.204	57,138	0.165 ± 0.216	55,381	0.131 ± 0.188
7	56,080	0.151 ± 0.200	56,256	0.158 ± 0.209	55,645	0.137 ± 0.194
8	54,964	0.164 ± 0.211	54,444	0.155 ± 0.206	54,142	0.133 ± 0.192
9	55,391	0.167 ± 0.218	54,938	0.170 ± 0.222	53,755	0.137 ± 0.196
10	57,388	0.168 ± 0.224	56,578	0.179 ± 0.230	56,003	0.148 ± 0.217
11	68,228	0.155 ± 0.198	66,998	0.155 ± 0.208	65,042	0.118 ± 0.177
12	58,985	0.165 ± 0.210	58,205	0.157 ± 0.207	57,224	0.131 ± 0.190
13	61,411	0.151 ± 0.204	60,698	0.170 ± 0.219	60,245	0.133 ± 0.197
14	67,767	0.133 ± 0.187	67,759	0.134 ± 0.188	67,623	0.114 ± 0.182
15	61,225	0.149 ± 0.202	61,225	0.153 ± 0.209	59,525	0.129 ± 0.189
16	57,537	0.149 ± 0.199	58,498	0.157 ± 0.212	56,483	0.124 ± 0.184
17	65,423	0.155 ± 0.208	64,034	0.152 ± 0.208	62,553	0.125 ± 0.191
18	60,008	0.149 ± 0.200	59,676	0.156 ± 0.205	59,039	0.128 ± 0.191
19	60,499	0.156 ± 0.213	61,012	0.167 ± 0.217	59,829	0.139 ± 0.201
20	57,307	0.150 ± 0.197	57,482	0.138 ± 0.187	56,644	0.117 ± 0.168
21	69,546	0.152 ± 0.206	68,146	0.151 ± 0.205	68,551	0.124 ± 0.175
22	57,485	0.154 ± 0.204	57,852	0.155 ± 0.209	56,128	0.125 ± 0.192
23	69,395	0.156 ± 0.193	68,358	0.140 ± 0.189	67,233	0.107 ± 0.164
24	71,118	0.134 ± 0.184	71,232	0.134 ± 0.187	70,438	0.108 ± 0.168
25	55,503	0.141 ± 0.190	56,750	0.142 ± 0.202	54,932	0.116 ± 0.183
26	61,598	0.143 ± 0.190	62,022	0.137 ± 0.178	59,862	0.116 ± 0.171
mean	60,421	0.155 ± 0.204	60,194	0.156 ± 0.208	59,062	0.128 ± 0.188

Valle del Belice (VDB), Comisana (COM) and Pinzirita (PIN) sheep breeds.

### Genetic diversity and population structure

Principal Component Analysis (PCA), Bayesian model-based clustering algorithm, calculation of *F*_ST_ and Neighbor network were used to visualize and explore the genetic relationships among breeds. Genotypes from seven additional sheep breeds belonging to International Sheep Genomics Consortium (Additional file 3: Table S3) were included in the analysis to place the Sicilian sheep breeds in a global context.

**Figure 2:**
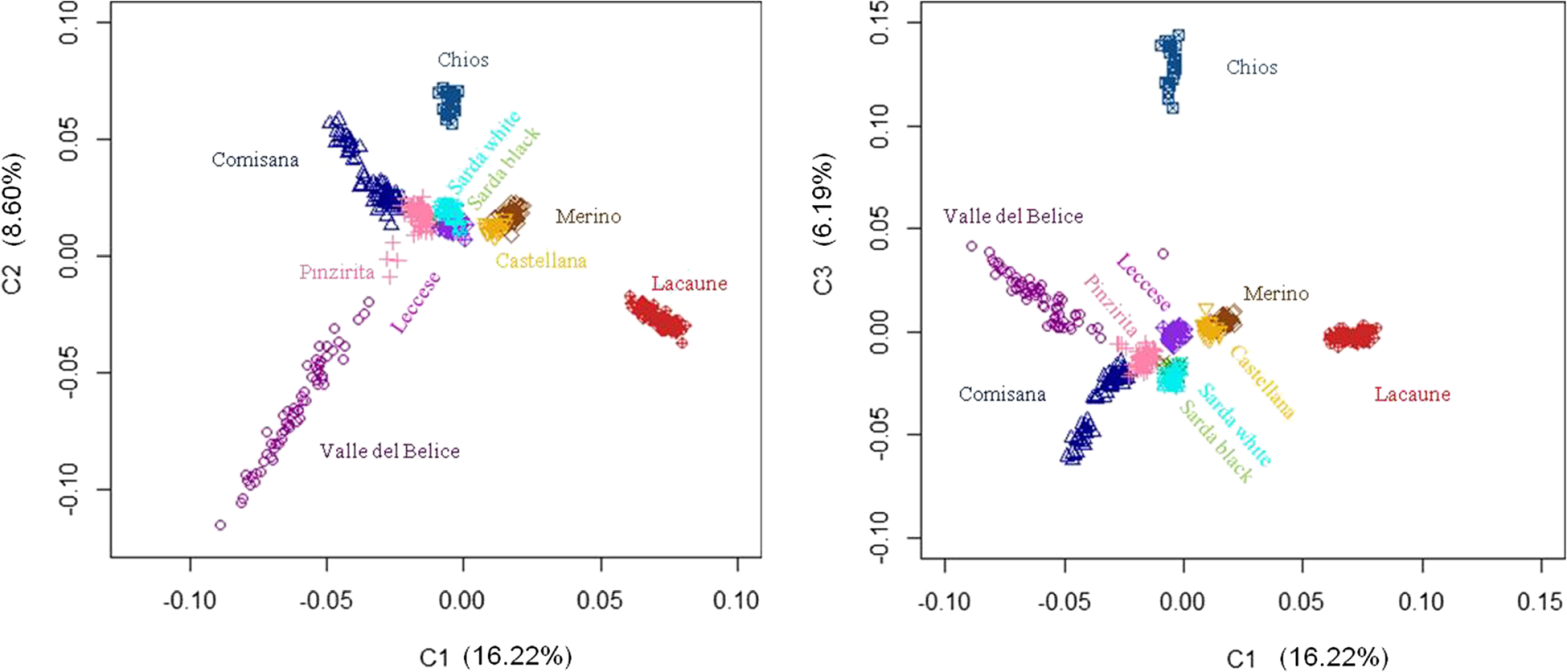
**Principal components analysis for the genetic differentiations among all sheep breeds using PC1 and PC2 (left), and PC1 and PC3 (right).**

After data editing, a total of 42,422 SNPs common to 486 individuals from 10 sheep breeds were analyzed. The PCA showed that most sheep breeds formed non-overlapping clusters and are clearly separated populations, except for Sarda white and Sarda black sheep breeds (Figure 2). Genetic similarity between the two breeds of Sardinia island was in agreement with previous studies [21]. Moreover, the PCA separated the breeds according to their geographic origin, with Italian breeds positioned apart from other European ones.

**Figure 3:**
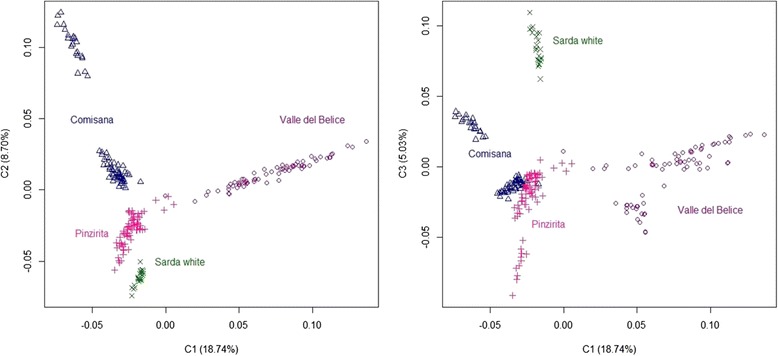
**Principal components analysis for the genetic differentiations between Sicilian and Sarda white sheep breeds using PC1 and PC2 (left), and PC1 and PC3 (right).**

To explore in detail the relatedness among Sicilian breeds, PCA was performed separately for Valle del Belice, Comisana and Pinzirita breeds. In addition to these autochthonous breeds, Sarda white was considered due to its likely contribution to the phylogenetic origin of Valle del Belice breed. While for Valle del Belice, Pinzirita and Sarda white breeds the two components (PC1 and PC2) clustered animals from the same breed together, the Comisana breed showed two groups (Figure 3). In fact, individuals from Comisana occupied different areas of the cluster, indicating the presence of substructure, and this could evoke concerns about the generation of false positive results when using LD mapping as the only mean to locate genes underlying complex traits [18]. The separation of Comisana individuals in these two sub-populations corresponded approximately to geographical areas of Sicily in which they were collected. Therefore, the genetic structure detected for Comisana sheep breed could be due to introgression of genes from other breeds and/or to geographical isolation of some farms for a long time. In fact, cross-breeding is known to be one of the main factors responsible for erosion of local breeds. The presence of substructure was confirmed by combination of PC1 and PC3 (Figure 3).

Moreover, some individuals of Comisana breed were positioned near the cluster of Pinzirita breed. The genetic closeness between these two breeds might be explained considering that they are characterized by a common breeding system and geographical husbandry area, which might have led to genetic exchange between them [22]. The relative contribution of SNPs to population assignment was estimated. In fact, high-throughput genotyping tools make it possible to extract interesting genetic information from animal populations that could be applied to identify useful markers for breed allocation, assignment parentage [23], and for tracing geographic origin of animal products [9] as meat and mono-breed cheese. Among the 44,610 SNPs common to the three Sicilian breeds, a subset of 119 SNPs was used to evaluate their ability to cluster individuals belonging to the Sicilian sheep breeds. These SNPs were selected considering their informativeness in pair comparisons that means SNPs with the larger allele frequency differences between pairs of breeds were chosen (fixed alleles in one breed and MAF > 0.25 in the other ones). In fact, differences in allele frequency provide the basis for assignment of individuals into discrete populations. PCA using this subset of SNPs showed lack of ability to discriminate among the breeds and the presence of overlapping areas, in particular between Comisana and Pinzirita sheep breeds (Additional file 4: Figure S1). Kijas et al. [7] revealed that while 96 of the most informative SNPs (higher average allelic richness and lower average private allelic richness) were insufficient, analysis using a panel of 384 markers successfully sorted individuals into groups. Our results may be due to close relationships among Sicilian sheep breeds that are genetically connected among them [22,24]. Therefore, the subset of SNPs was not useful to assign individuals into discrete clusters and it was insufficient and inadequate for authentication purposes.

**Figure 4:**
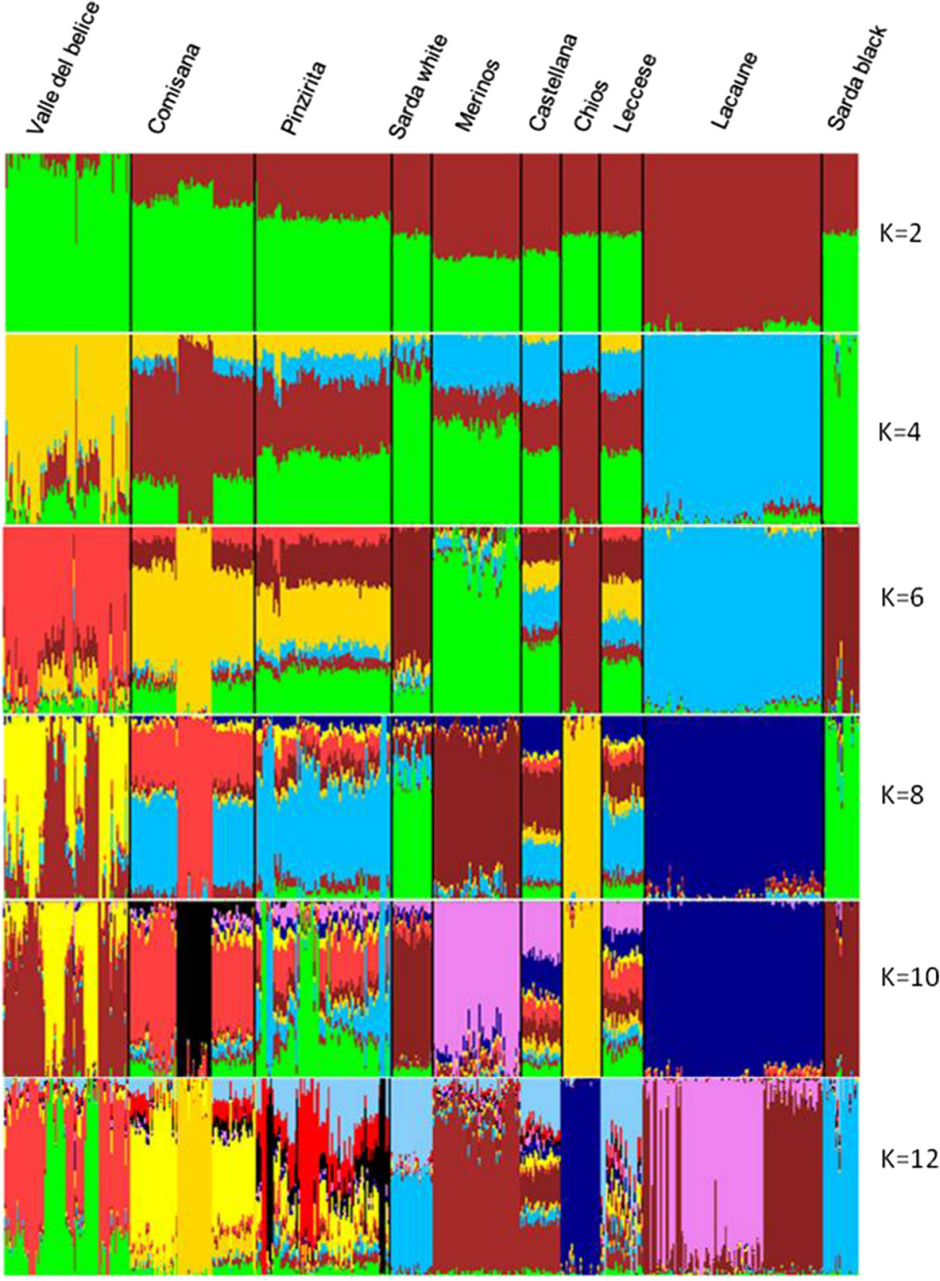
**Model based clustering of the estimated membership fractions of individuals of the 10 breeds analyzed in each of the K inferred clusters, for K = 2 to K = 12.**

**Figure 5:**
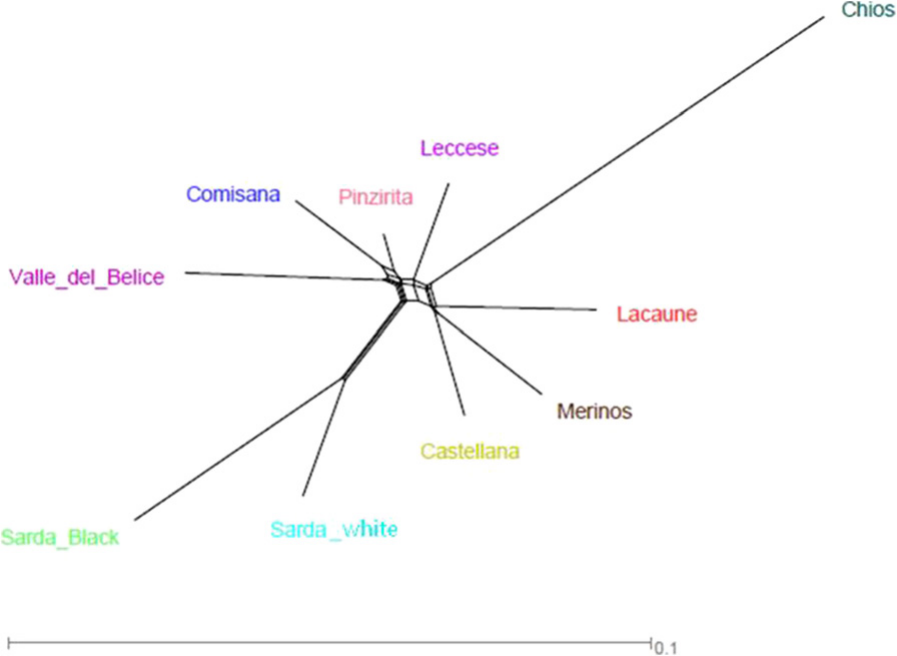
**Relationship between breeds based on Neighbor network obtained using pair-wise estimates of** ***F***_**ST.**_ **.**

Results from within population substructure through Admixture analysis, considering a range of 2 through 20 potential clusters (K), pointed out that the best fitting number of populations present in the total sample was K = 12. A graphic representation of the estimated membership coefficients to the 12 clusters is shown in Figure 4, where model-based clustering partitioned the genome of each sample into a predefined number of components [25]. The first breeds to be differentiated from the others were Lacaune and Valle del Belice (K = 2). Other breed-specific clusters were Chios and Merinos (K = 6); at K = 8 some genomic components appeared to be shared by several breeds, as for example between Comisana and Pinzirita, whereas at K = 10, each breed tends to have its own distinct cluster but with some differences; in fact, Sicilian breeds showed less distinct clusters than other breeds as Lacaune, Chios and Sarda black. For some breeds, as the two from Sardinia island, the admixture analysis revealed a shared ancestry; in fact these breeds clustered together at high K value (K = 10). These results reflect geographic proximity and confirmed the findings based on the PCA, where, for example, Chios and Lacaune formed separated and differentiated clusters. For the Sicilian breeds, results were in agreement with a previous study conducted on the genetic structure and relationship using microsatellite markers [22] that showed low genetic differentiation and high level of admixture among Valle del Belice, Comisana, and Pinzirita breeds. Moreover, at K = 10 the results evidenced again that Comisana is split in two mixed groups. The degree of genetic differentiation between pairs of breeds was showed in Table 2. Considering pairwise *F*_ST_ among all populations, Chios was the most divergent breed. The highest values were observed between Chios and both Sarda breeds (*F*_ST_ = 0.139 and 0.120 for Sarda black and Sarda white, respectively), while the lowest one was observed between Comisana and Pinzirita breeds (*F*_ST_ = 0.025). These results may be explained considering the geographic origin of the breeds: Chios and the two Sarda breeds belonging to different countries, whereas Comisana and Pinzirita were two breeds reared in the same island. The *F*_ST_ value between pairs of breeds was also used to reconstruct the NeighborNet graph (Figure 5), showing some clear clusters and relationships between breeds that originated from the same regions: Sicilian, Sardinian, and European breeds. The shortest branch was observed for Pinzirita breed, while the longest one was found for Chios breed that was the most differentiated and isolated population among the analyzed breeds. Considering only the Sicilian breeds, Valle del Belice showed the longest branch, while Pinzirita the shortest one, according to the results of genetic diversity for these breeds (Table 3). In fact, Kijas et al. [26] in a study on genome-wide analysis of the world’s sheep breeds showed short branches for breeds with high heterozygosity, while long branches for breeds with low heterozygosity. The topology of the NeighborNet constructed with *F*_ST_ distances between breeds perfectly coincides with PCA (Figure 2). NeighborNet graph was also depicted considering the substructure present in Comisana breed (Additional file 5: Figure S2). The figure showed that the two sub-populations originated from the same branch and displayed a very close relationship, which was expected considering that belonging to the same breed. The reticulations towards the extremity of the graphs indicated increasing genetic relatedness between breeds. In fact, NeighborNet provide a robust framework for inferring and investigating phylogenetic networks.

**Table 2:** **Genetic differentiation between population pairs measured using** ***F***_**st**._

	**PIN**	**COM**	**SAB**	**VDB**	**CHI**	**CAS**	**MER**	**SAW**	**LAC**	**LEC**
PIN	0									
COM	0.025	0								
SAB	0.065	0.080	0							
VDB	0.041	0.051	0.090	0						
CHI	0.086	0.096	0.139	0.112	0					
CAS	0.033	0.045	0.078	0.059	0.096	0				
MER	0.038	0.051	0.080	0.063	0.099	0.038	0			
SAW	0.046	0.060	0.059	0.071	0.120	0.060	0.061	0		
LAC	0.041	0.054	0.083	0.066	0.103	0.044	0.048	0.064	0	
LEC	0.030	0.040	0.075	0.054	0.095	0.038	0.043	0.054	0.047	0

(PIN = Pinzirita, COM = Comisana, SAB = Sarda black, VDB = Valle del Belice, CHI = Chios, CAS = Castellana, MER = Merino, SAW = Sarda white, LAC = Lacaune, LEC = Leccese).

**Table 3:** **Estimates of genetic diversity indices for Sicilian sheep breeds**

**Breeds**	**MAF ± s.d.**	**Ho ± s.d.**	**He ± s.d.**	***F*** **± s.d.**	**N**_**e**_
Valle del Belice	0.290 ± 0.003	0.364 ± 0.126	0.379 ± 0.155	0.055 ± 0.150	369
Comisana	0.294 ± 0.004	0.382 ± 0.129	0.382 ± 0.114	0.025 ± 0.031	400
Pinzirita	0.301 ± 0.005	0.388 ± 0.122	0.390 ± 0.108	0.016 ± 0.042	685

Average minor allele frequency (MAF), observed heterozygosity (Ho), expected heterozygosity (He), inbreeding coefficient (*F*) and standard deviation (s.d.), and effective population size (N_e_).

Understanding the relationships among and within populations is an important step to establish conservation priorities and strategies. Consistency of results among the several used approaches (PCA, Bayesian clustering, *F*_ST,_ Neighbor networks) supported the robustness of our conclusions.

### Estimates of the genetic diversity in Sicilian sheep breeds

The basic genetic diversity indices within breed were used to compare levels of heterogeneity between Sicilian breeds. Overall average MAF was 0.290 ± 0.003 for Valle del Belice, 0.294 ± 0.004 for Comisana, and 0.301 ± 0.005 for Pinzirita sheep breeds (Table 3). These values were in agreement with those reported by Kijas et al. [26] in a study on genome-wide analysis of the world’s sheep breeds for the European-derived populations. Moreover, the distribution of MAF of these SNPs was approximately uniform over the genome in all breeds (Additional file 1: Table S1). Pinzirita breed displayed the highest gene diversity (He = 0.390 ± 0.108), whereas the lowest value was found in Valle del Belice breed (He = 0.379 ± 0.155) (Table 3). These genetic diversity estimates can be compared with those reported by other authors for Southern and Mediterranean European sheep breeds [26]. Similar results for genetic diversity (Ho, He, and MAF) were reported for Sarda sheep breed [14]. We obtained some negative values for inbreeding coefficient *F*, which corresponded to animals with lower homozygosity than the average population. The highest *F*, calculated for each individual based upon observed and expected heterozygosity, was found in Valle del Belice breed (0.055 ± 0.150), whereas the lowest value in Pinzirita breed (0.016 ± 0.042). In Valle del Belice breed, the high level of genetic differentiation within breed, with individuals spread out on PC1 (Figure 3), and heterogeneous cluster in Admixture analysis (Figure 4), but at the same time the highest inbreeding coefficient (Table 3), suggested that the breed had a wide genetic base with inbred individuals belonging to the same flock. Rosa et al. [27] in a study on parentage verification of Valle del Belice breed, reported different values of inbreeding per flock that ranged from 0.017 to 0.165. In fact, in Sicilian farming system natural mating is the common practice, the exchange of rams among flocks is quite unusual, and mating with close relatives can be quite frequent; this led to high level of inbreeding. Analogous results for Valle del Belice breed were reported by Tolone et al. [22]. Therefore, the results (high level of genetic differentiation and high inbreeding coefficient) may be explained considering reduced or absent gene flow between different flocks. It should be noted that Valle del Belice was the most homogeneous breed at the lowest K values, according with higher inbreeding and lower heterozygosity. Moreover, the low level of inbreeding and high genetic diversity in Pinzirita sheep breed reflected the short extent of LD. Average values of LD for 1 Mb bins smoothed with a 50 kb step size were used to estimate the effective population size (N_e_). In fact, LD is affected by population history and demography, representing an important tool to be applied to genetic population. Effective population size (N_e_) is a general indicator of the risk of genetic erosion, contains relevant information for the monitoring of the genetic diversity, and helps to explain how populations evolved [28]. The highest value of N_e_, estimated 50 generations ago, was observed for Pinzirita breed (N_e_ = 685), whereas the lowest one was observed for Valle del Belice breed (N_e_ = 369) (Table 3). In fact, high N_e_ is accompanied with high genetic diversity and low values of LD. Moreover, it is important to remind that LD measures, and therefore N_e_, are linked with selection intensity and mating systems. Indeed, high selection pressure and the use of artificial insemination are the main reasons for low N_e_ values. Ciani et al. [21] in a study on Italian sheep breeds reported similar results of N_e_ in Sicilia sheep breeds with 638, 571, and 340 individuals for Pinzirita, Comisana, and Valle del Belice breeds, respectively. Moreover, García-Gámez et al. [1] in Churra sheep breed reported N_e_ estimated 50 generation ago of 467 individuals. Our results were consistent with those reported for most European sheep breeds [26] that showed high N_e_. Managing the N_e_ and *F* provides a general framework to control loss of variability avoiding or alleviating reductions in viability and fertility; i.e., inbreeding depression [29].

## Conclusions

This study reported for the first time estimates of linkage disequilibrium, genetic diversity and population structure from a genome-wide perspective in Sicilian dairy sheep breeds. Knowledge concerning the behavior of LD is important for performing genomic selection and genome wide association analysis. LD declined as a function of distance and average *r*^*2*^ was lower than value observed in other sheep breeds. Results indicated that Sicilian sheep breeds formed non-overlapping clusters, with low genetic differentiation and high level of admixture, and that the Comisana breed does not constitute a homogenous population. The different approaches (PCA analysis, Bayesian clustering, *F*_ST,_ NeighborNet) used to assess relationships among breeds showed high degree of congruence and supported the robustness of conclusions. Analysis of genetic diversity indicated high genetic variability and low inbreeding. The identification of genetic relationship and gene flow among livestock breeds/populations is important for breeders and conservationists. In order to maintain the existing genetic diversity, breeding strategies aiming at maintaining effective population size, minimizing inbreeding and genetic drift should be implemented for the Sicilian breeds. The information generated from this study can have important implications for the design and applications of association studies as well as for the development of conservation and/or selection breeding programs.

## Methods

### DNA sampling and genotyping

A total of 221 unrelated animals were collected from several farms in different areas of Sicily, to capture a representative sample of within breed genetic diversity, and were used for the analysis.

The procedures involving animal sample collection followed the recommendation of directive 2010/63/EU. Number of animal sampled per flock ranged from 5 to 10. Samples consisted of 72 Valle del Belice, 72 Comisana and 77 Pinzirita individuals. For these sheep breeds, pedigree data were not available. About 10 ml of blood was collected from jugular vein using tubes with EDTA as anticoagulant. Genomic DNA was extracted from buffy coats of nucleated cells using salting out method [30]. The concentration of extracted DNA was assessed with NanoDrop ND-1000 spectrophotometer (NanoDrop Technologies, Wilmington, DE).

All animals were genotyped for 54,241 SNPs, using the Illumina OvineSNP50K Genotyping BeadChip following standard operating procedures recommended by the manufacturer. Genotyping was performed by Dipartimento Scienze Agrarie e Forestali, University of Palermo. Raw signal intensities were converted into genotype calls using the Illumina GenomeStudio Genotyping Module v1.0 software (Illumina Inc., San Diego, CA) by applying a no-call threshold of 0.15. Genotyping data were initially tested for quality using the same software. Markers in each breed were filtered to exclude loci assigned to unmapped contigs. Therefore, only SNPs located on autosomes were considered in further analyses. Moreover, quality control included: Call Frequency (proportion of samples with genotype at each locus) ≥ 0.95, minor allele frequency (MAF) ≥ 0.05, and Hardy-Weinberg Equilibrium (HWE) *P*-value > 0.001. SNPs that did not satisfy these quality criteria were discarded.

### Linkage Disequilibrium

A standard descriptive Linkage Disequilibrium (LD) parameter, the squared correlation coefficient of allele frequencies at pair of loci (*r*^*2*^), was used as measure. Pairwise LD between adjacent SNPs was calculated on each chromosome using PLINK [31]. Moreover, *r*^*2*^ was estimated for all pairwise combinations of SNPs using LD plot function in Haploview v4.2 software [32], exporting data to text files. For each chromosome, pairwise *r*^*2*^ was calculated for SNPs between 0 and 50 Mb apart. To visualize the LD pattern per chromosome, *r*^*2*^ values were stacked and plotted as a function of inter-marker distance categories. Average *r*^*2*^ for SNP pairs in each interval was estimated as the arithmetic mean of all *r*^*2*^.

### Analysis of genetic diversity and population structure among breeds

In order to understand the genetic relationship among Sicilian breeds, we performed different analyses. Genotypes from others 7 sheep breeds belonging to International Sheep Genomics Consortium (Additional file 2: Table S2), were included in these analyses. Genotyping data from Sicilian and other breeds were filtered with the same quality criteria reported above. Unlinked SNPs were selected using *-indep* option of the PLINK [31], to reduce the impact of the SNP ascertainment bias phenomenon, with the following parameters: 50 SNPs per window, a shift of 5 SNPs between windows, and a variation inflation factor’s threshold of 2.

First, the average proportion of alleles shared between animals (*A*_*s*_) was calculated as IBS2 + 0.5*IBS1/N, where IBS1 and IBS2 are the number of loci that share either one or two alleles identical by state (IBS), respectively, and N is the number of loci tested. Genetic distance (*D*) was calculated as 1-*A*_*s*_. These values were calculated using PLINK [31] through the use of commands *-cluster* and *-distance-matrix*. PCA of *D* matrix was performed using the multidimensional scaling (MDS) algorithm of pairwise genetic distance implemented in PLINK [31]. It should be noted that when MDS is applied to *D* matrix, it is numerically identical to PCA [31]. The graphical representation was depicted using the statistical *R* software (R Development Core Team) with RColorBrewer package.

Extents of population substructure were evaluated through the model-based clustering algorithm implemented in the software Admixture [33]. The most probable number of populations in the dataset (K) was estimated using the default (5-fold) Admixture’s cross-validation procedure, by which estimated prediction errors are obtained, for each K value, by adopting a kind of ‘leave one-out’ approach. K value that minimizes the estimated prediction errors is then assumed to be the most suitable. Graphical representation was visualized using the statistical *R* software (R Development Core Team).

Genepop [34] software was used to estimate population relatedness using pair-wise estimates of *F*_ST_ among all breeds. Neighbor networks were constructed from pair-wise estimates of *F*_ST_ using Splitstree [35].

### Estimates of the genetic diversity in Sicilian sheep breeds

PLINK [31] was also used to estimate basic genetic diversity indices, including observed and expected heterozygosity (Ho and He, respectively), average MAF and coefficient of inbreeding (*F*) for Sicilian sheep breeds. Files used for basic diversity indices (Ho, He and *F*) were pruned in PLINK considering 50 SNPs per windows, a shift of 10 SNPs between windows and variation inflation factor’s threshold of 1.5. Moreover, effective population sizes (N_e_) were calculated as N_e_ = (1/4*c*)*(1/ *r*^*2*^-1) [36], where *r*^*2*^ is the value of LD and *c* is the marker distance in Morgans between SNPs.

## Additional files

Additional file 1: Table S1. Average MAF (Minor Allele Frequency) per chromosome (OAR) and number of SNPs (N°) that passed quality control in the Sicilian sheep breeds. Valle del Belice (VDB), Comisana (COM), and Pinzirita (PIN) sheep breeds. Additional file 2: Table S2. Mean Linkage Disequilibrium (*r* ^*2*^) among SNPs over different map distances in Sicilian sheep breeds. Valle del Belice (VDB), Comisana (COM), and Pinzirita (PIN) sheep breeds. Additional file 3: Table S3. Number of sampled animals from seven additional sheep breeds. Additional file 4: Figure S1. Principal components analysis between Sicilian breeds using marker panel contained 119 SNPs. Valle del Belice (VDB), Comisana (COM), and Pinzirita (PIN) breeds. Additional file 5: Figure S2. Neighbor network obtained using pair-wise estimates of *F*_ST_ between breeds, considering the sub-populations in Comisana breed.
