# Supplementary material for: Genome wide linkage disequilibrium and genetic structure in Sicilian dairy sheep breeds
Source: BMC Genet. 2014 Oct 10;15:108. doi: 10.1186/s12863-014-0108-5 (PMC4197223; doi:10.1186/s12863-014-0108-5)
Supplement: Additional file 1: Table S1. — Average MAF (Minor Allele Frequency) per chromosome (OAR) and number of SNPs (N°) that passed quality control in the Sicilian sheep breeds. Valle del Belice (VDB), Comisana (COM), and Pinzirita (PIN) sheep breeds. [file 12863_2014_108_MOESM1_ESM.doc]

**Table S1 Average MAF (Minor Allele Frequency) per chromosome (OAR) and number of SNPs (N°) that passed quality control in the Sicilian sheep breeds.** Valle del Belice (VDB), Comisana (COM) and Pinzirita (PIN) sheep breeds.

|  | **VDB** | | **COM** | | **PIN** | |
| --- | --- | --- | --- | --- | --- | --- |
| **OAR** | **MAF** | **N°** | **MAF** | **N°** | **MAF** | **N°** |
| 1 | 0.293 | 4,962 | 0.298 | 4,976 | 0.303 | 5,089 |
| 2 | 0.286 | 4,650 | 0.291 | 4,615 | 0.300 | 4,759 |
| 3 | 0.285 | 4,225 | 0.291 | 4,253 | 0.298 | 4,357 |
| 4 | 0.287 | 2,294 | 0.288 | 2,334 | 0.295 | 2,365 |
| 5 | 0.289 | 2,008 | 0.293 | 2,025 | 0.300 | 2,064 |
| 6 | 0.287 | 2,204 | 0.291 | 2,208 | 0.300 | 2,278 |
| 7 | 0.300 | 1,921 | 0.298 | 1,915 | 0.305 | 1,936 |
| 8 | 0.294 | 1,780 | 0.297 | 1,797 | 0.305 | 1,807 |
| 9 | 0.287 | 1,819 | 0.292 | 1,834 | 0.299 | 1,876 |
| 10 | 0.291 | 1,539 | 0.294 | 1,561 | 0.299 | 1,577 |
| 11 | 0.271 | 981 | 0.289 | 999 | 0.294 | 1,029 |
| 12 | 0.293 | 1,440 | 0.293 | 1,460 | 0.297 | 1,485 |
| 13 | 0.294 | 1,448 | 0.285 | 1,465 | 0.300 | 1,476 |
| 14 | 0.292 | 9,96 | 0.286 | 997 | 0.302 | 999 |
| 15 | 0.291 | 1,439 | 0.292 | 1,439 | 0.298 | 1,462 |
| 16 | 0.287 | 1,340 | 0.286 | 1,318 | 0.296 | 1,365 |
| 17 | 0.290 | 1,200 | 0.296 | 1,226 | 0.305 | 1,255 |
| 18 | 0.291 | 1,199 | 0.291 | 1,205 | 0.300 | 1,218 |
| 19 | 0.292 | 1,072 | 0.297 | 1,063 | 0.296 | 1,084 |
| 20 | 0.298 | 945 | 0.288 | 945 | 0.300 | 960 |
| 21 | 0.299 | 760 | 0.293 | 774 | 0.311 | 771 |
| 22 | 0.285 | 918 | 0.283 | 914 | 0.296 | 942 |
| 23 | 0.283 | 955 | 0.298 | 970 | 0.307 | 987 |
| 24 | 0.290 | 623 | 0.291 | 622 | 0.299 | 629 |
| 25 | 0.285 | 866 | 0.294 | 847 | 0.296 | 875 |
| 26 | 0.293 | 781 | 0.300 | 778 | 0.299 | 806 |
|  | **0.290** | **44,365** | **0.292** | **44,540** | **0.300** | **45,451** |
