# Supplementary material for: Genome wide linkage disequilibrium and genetic structure in Sicilian dairy sheep breeds
Source: BMC Genet. 2014 Oct 10;15:108. doi: 10.1186/s12863-014-0108-5 (PMC4197223; doi:10.1186/s12863-014-0108-5)
Supplement: Additional file 2: Table S2. — Mean Linkage Disequilibrium (r 2) among SNPs over different map distances in Sicilian sheep breeds. Valle del Belice (VDB), Comisana (COM), and Pinzirita (PIN) sheep breeds. [file 12863_2014_108_MOESM2_ESM.doc]

**Table S2 Mean Linkage Disequilibrium (*r^2^*) among SNPs over different map distances in Sicilian sheep breeds.** Valle del Belice (VDB), Comisana (COM) and Pinzirita (PIN) sheep breeds.

| **Distance range (kb)** | ***r^2^* (VDB)** | ***r^2^*(COM)** | ***r^2^*(PIN)** |
| --- | --- | --- | --- |
| **<50** | 0.183 | 0.181 | 0.154 |
| **50-100** | 0.116 | 0.113 | 0.084 |
| **100-200** | 0.089 | 0.085 | 0.056 |
| **200-500** | 0.074 | 0.067 | 0.041 |
| **500-1,000** | 0.066 | 0.061 | 0.036 |
| **1,000-2,000** | 0.058 | 0.055 | 0.034 |
| **>2,000** | 0.041 | 0.042 | 0.029 |
