# Supplementary material for: Genome wide linkage disequilibrium and genetic structure in Sicilian dairy sheep breeds
Source: BMC Genet. 2014 Oct 10;15:108. doi: 10.1186/s12863-014-0108-5 (PMC4197223; doi:10.1186/s12863-014-0108-5)
Supplement: Additional file 3: Table S3. — Number of sampled animals from seven additional sheep breeds. [file 12863_2014_108_MOESM3_ESM.doc]

| Breed | Number of individuals |
| --- | --- |
| Sarda white | 24 |
| Sarda black | 20 |
| Leccese | 24 |
| Castellana | 23 |
| Chios | 23 |
| Merino | 50 |
| Lacaune | 103 |

**Table S3.** Number of sampled animals from seven additional sheep breeds
