# Supplementary figures and images for: Genome wide linkage disequilibrium and genetic structure in Sicilian dairy sheep breeds
Source: BMC Genet. 2014 Oct 10;15:108. doi: 10.1186/s12863-014-0108-5 (PMC4197223; doi:10.1186/s12863-014-0108-5)

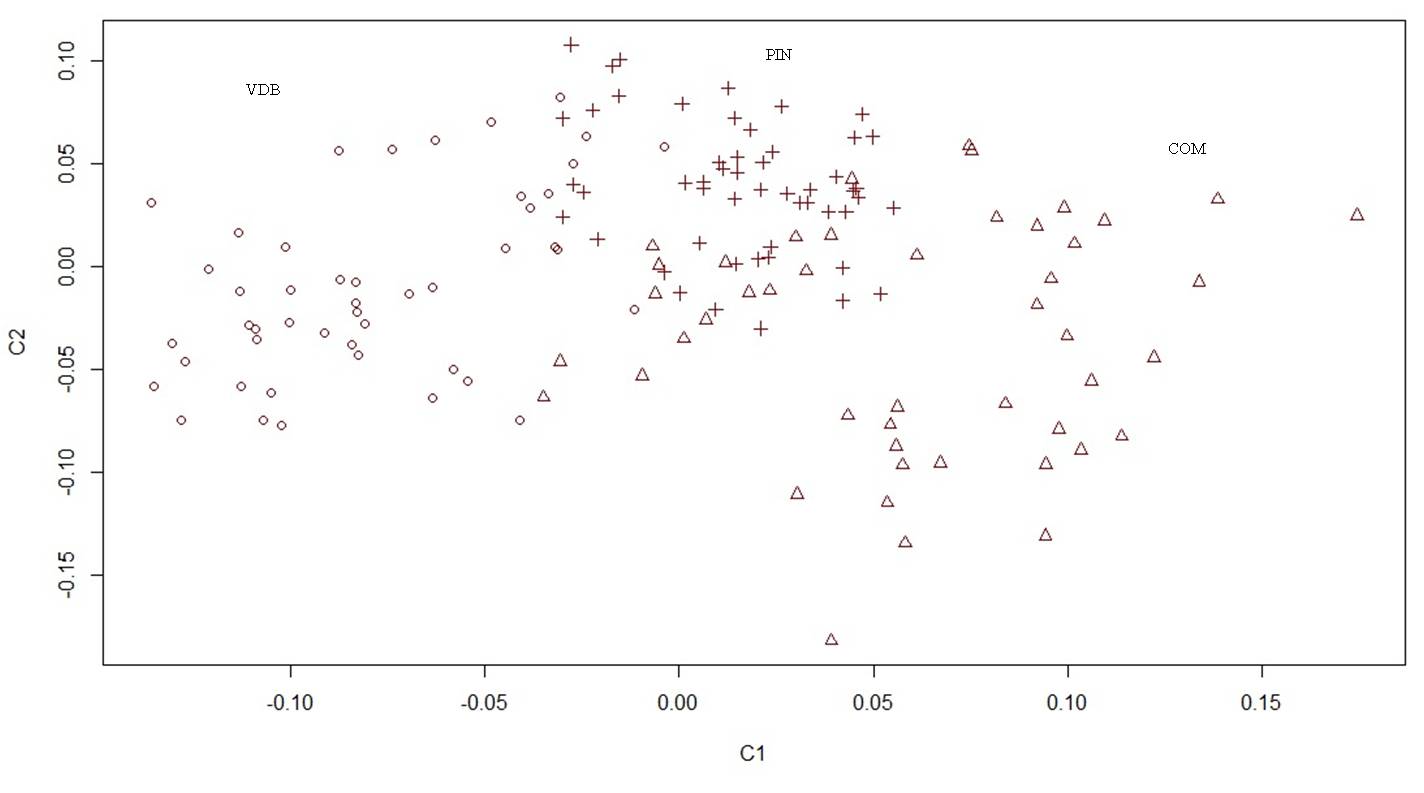

Supplement: Additional file 4: Figure S1. — Principal components analysis between Sicilian breeds using marker panel contained 119 SNPs. Valle del Belice (VDB), Comisana (COM), and Pinzirita (PIN) breeds. [file 12863_2014_108_MOESM4_ESM.jpeg]

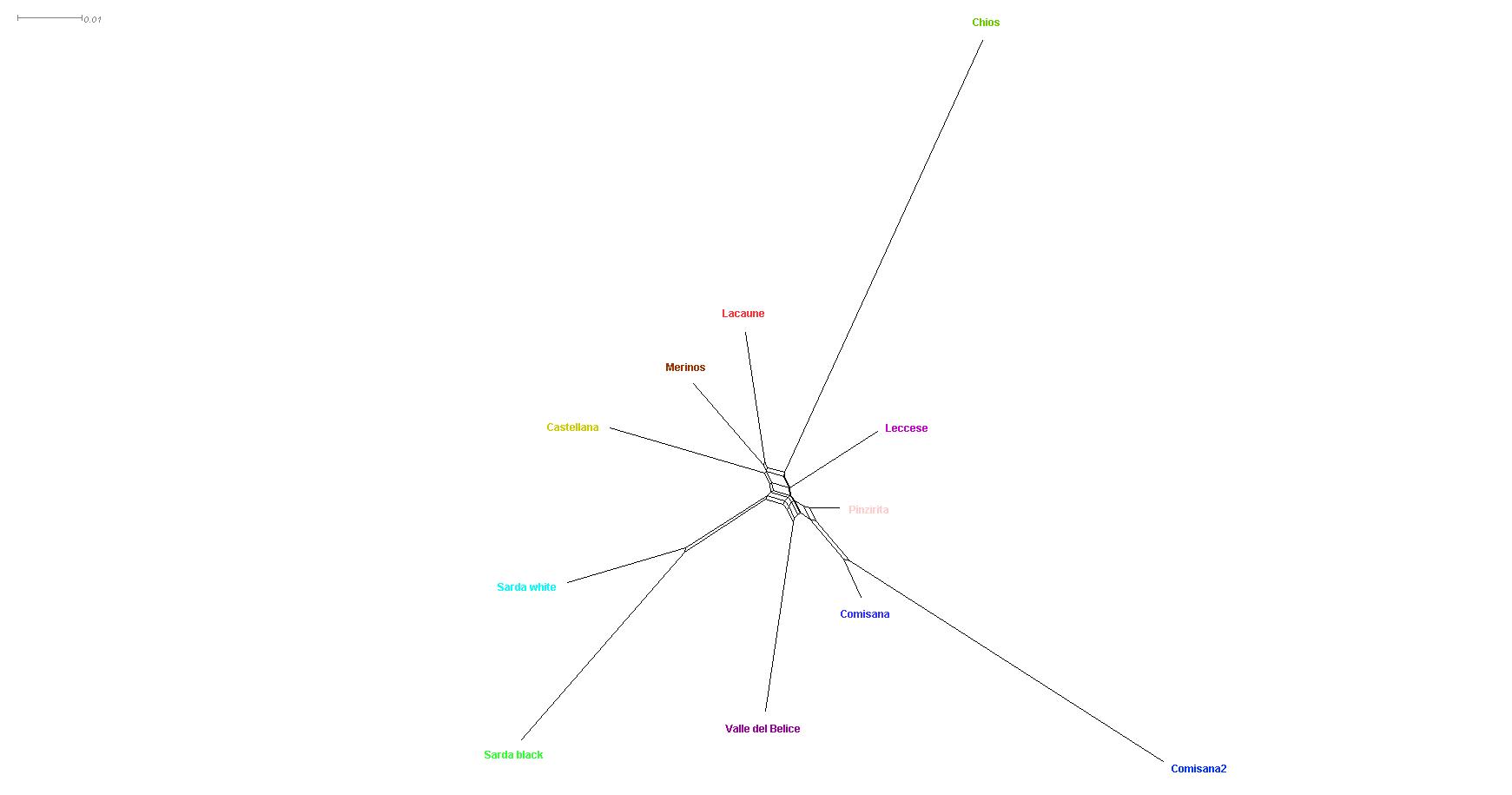

Supplement: Additional file 5: Figure S2. — Neighbor network obtained using pair-wise estimates of F ST between breeds, considering the sub-populations in Comisana breed. [file 12863_2014_108_MOESM5_ESM.jpeg]
